# Effect of Surface Roughness on the Electrochemical Behavior and Corrosion Resistance of TiTaNbZrAg Alloy with Different Amounts of Tantalum in Bulk Composition

**DOI:** 10.3390/ma17215217

**Published:** 2024-10-26

**Authors:** Gabriel Dobri, Alexandra Banu, Cristina Donath, Elena Ionela Neacsu, Mihai Anastasescu, Monica Elisabeta Maxim, Cora Vasilescu, Loredana Preda, Maria Marcu

**Affiliations:** 1 Surface Engineering and Corrosion Laboratory, Faculty of Industrial Engineering and Robotics, National University of Science and Technology Politehnica Bucharest, Splaiul Independentei 313, 060042 Bucharest, Romania; gabriel.dobri@upb.ro (G.D.); alexandra.banu@upb.ro (A.B.); 2 Institute of Physical Chemistry-Ilie Murgulescu, Splaiul Independentei 202, 060021 Bucharest, Romania; cdonath@icf.ro (C.D.); eneacsu@icf.ro (E.I.N.); manastasescu@icf.ro (M.A.); mmaxim@icf.ro (M.E.M.); cvasilescu@chimfiz.icf.ro (C.V.)

**Keywords:** titanium alloys, corrosion, biomaterials, PBS, surface roughness

## Abstract

The corrosion behavior of the TiTaNbZrAg alloys with different amounts of tantalum (0%, 10% and 20%) and with distinct surface topography (smooth and rough) was investigated in phosphate buffer solution (PBS) for long-time immersion (1000 h). By this approach, we expect to bring about new insights into the influence of both the amount of Ta in the alloy composition and the surface topography on the corrosion behavior of the Ti-based alloys. The corrosion resistance was studied by Open Circuit Potential (OCP), Potentiodynamic Polarization (PP), and Electrochemical Impedance Spectroscopy (EIS). From the potentiodynamic investigations, it was observed that all types of samples showed good corrosion resistance (i.e., *R_corr_* < 10 µm y^−1^) and may be used successfully for medical applications. However, the samples with smooth surfaces and with a certain amount of Ta (10% and 20%) exhibit the best corrosion performance (*R_corr_* < 1 µm y^−1^). As regards the samples with rough surfaces, the results evidenced that they showed lower corrosion resistance (1 < *R_corr_* < 3 µm y^−1^), suggesting that the Ta presence does not necessarily hinder the corrosion processes. Actually, the synergetic effect of both the presence of Ta and surface roughness plays an important role in corrosion resistance.

## 1. Introduction

Cobalt-chromium alloys, magnesium-based alloys, stainless steel and pure titanium, were materials regularly utilized for medical applications [1,2]. Intensive research carried out on these alloys attested that they have relatively poor corrosion resistance and mechanical properties. As an alternative to these alloys, growing attention was paid to the Ti-based alloys containing different alloying elements, like cpTi and TiNi [3,4]. Titanium-based alloys are frequently used in the medical field due to their excellent corrosion performance, high biocompatibility, long-term functionality and relatively low Young’s modulus [5,6]. As ion release measurements showed that these alloying elements are toxic [7], in recent years, a growing concern was about obtaining new titanium alloys without alloying elements that have toxic side effects on long-term applications; for instance, Ti-6Al-4V has a Young’s modulus not really close to the human bone. From electrochemical and ion release analysis, it was evident that these alloys have, for instance, a certain instability, making them limited for biomedical applications. This instability most probably relies on the presence of a higher amount of α phase, which is well known as being less stable. Therefore, in order to overcome this issue, recent research works were devoted to the development of a new group of titanium alloys like Ti–Nb-Zr-Ta-based alloys that contain nontoxic majority β-phase stabilizers, tantalum, niobium and zirconium [8,9,10]. It appears that at these types of alloys, the presence of Ta, Nb and Zr alloying elements in the Ti-based alloy composition yields the majority of β-Ti phase structure, providing hence it Young’s modulus close to that of human bone (60–80 GPa), mechanical strength, high corrosion resistance and good biocompatibility [11,12]. This high corrosion resistance is mainly provided by the presence of Ta, which generates a thick oxide film spontaneously formed on the surface that protects the alloy against biological corrosion and simultaneously facilitates the deposition of apatite coating in simulated body fluid (SBF) which further accelerates the adherence of osseous tissues [6]. Besides, the content of Ta in alloy composition seems to influence the corrosion behavior of these Ti-based alloys. Therefore, the minimum of Ta necessary for obtaining alloys with good corrosion behavior is around 5% [13], as observed for binary alloys (e.g., Ti-Ta) [14]. The increase in Ta content to 10% appears to slightly decrease the corrosion performance of these binary alloys. Since the content of Ta seems to influence the corrosion behavior of these Ti-based alloys, we studied the corrosion performance of Ti-Ta-Nb-Zr-Ag-alloys with different amounts of Ta (0–20%).

However, its presence as an alloying element in titanium alloys could cause serious issues like inhomogeneity in the structure of the alloy [14]. In order to overcome this problem, adding Nb and Zr as alloying elements proved to be a conspicuous solution. The antibacterial character of the alloy used for different medical applications should not be disregarded, and consequently, adding a slight amount of silver as alloying element proved to be a reliable solution.

Another important aspect that should herein be discussed is the biocompatibility of these alloys. A high biocompatibility facilitates the adhesion of the osteoblasts in biofluids and improves the osseointegration process of the implants [15,16]. Their biocompatibility relies on their structural properties, such as surface roughness, wettability, and mechanical and chemical properties [17,18]. For instance, the best cellular adhesion [19,20] was noticed for the implant surface, which shows minimal to moderate roughness and moderate hydrophilicity (i.e., about 60 Degrees). Since the surface topography appears to play an important role in the osseointegration process of the implants, carrying out studies about the influence of the surface roughness on the physio-chemical properties (like wettability and corrosion resistance) of the alloy is imperative for obtaining alloys with peculiar properties for medical applications. In literature was reported a high corrosion performance for titanium-based alloys with different amounts of Ta in composition and with smooth surfaces obtained by different technologies (cast, forging, arc-melting, etc.) [21,22] in several biofluids (e.g., Ringer’s or Hank’s solutions, artificial saliva or phosphate buffer solution (PBS)) for long-term immersion [23,24]. Although an alloy with a smooth surface ensures high corrosion performance, its use as an implant is limited because, for growing tissues on the surface implants, a certain roughness of the surface alloy is necessary. In other words, a rough surface develops a large area, which is desirable for the osseointegration process but, according to different reports, appears to affect the corrosion behavior of the biomaterial. In order to obtain the predetermined roughness of the implant surface, several methods of surface treatment (chemical treatment, sandblasting, mechanical grinding) were employed [25,26]. In our case, we used mechanical grinding to obtain different surface roughness within the limits of nanometrics, accepted for this kind of application [17]. The advantage of this technique relies on the fact that compared to others, it does not induce significant changes in the mechanical and structural properties of the alloy.

In spite of the fact that both the amount of Ta and surface roughness play important roles in the corrosion resistance of these alloys, scarce literature data were devoted to an ample study on the influence of both the amount of tantalum in the alloy composition and surface roughness on the corrosion behavior of these titanium-based alloys in different biofluids.

Based on this previous information, the physicochemical properties of as-cast TixTa9Nb8Zr2Ag alloys, after long periods of immersion (i.e., 1000 h) in PBS at 37 °C, with different amounts of Ta (0%, 10% and 20%) and different surface roughnesses were investigated for further possible applications in medical fields (e.g., implants). Previously [27,28], we studied the mechanical properties of these alloys, and we observed that their Young’s modulus is closer to that one of human bone (i.e., 82 GPa for 10% Ta and 55 GPa for 20% Ta), making them from a mechanical point of view suited for implant applications. The morphology and roughness of the surface alloys were analyzed before and after immersion in PBS by Atomic Forse Microscopy (AFM). The wettability of these alloys was studied by contact angle measurement. The corrosion behavior of our samples was investigated using several electrochemical methods [i.e., Open Circuit Potential (OCP), Potentiodynamic Polarization (PP) and Electrochemical Impedance Spectroscopy (EIS)]. The influence of chemical composition and surface roughness on the protective properties against corrosion attacks of these alloys are discussed in detail. It should be underlined that, in our case, using PBS as biofluid is appropriate because its pH (7.4) is similar to that of human blood and is nontoxic to living cells [29].

## 2. Materials and Methods

### 2.1. Sample Preparation

The Ti9Nb8Zr2Ag, Ti20Ta9Nb8Zr2Ag and Ti10Ta9Nb8Zr2Ag alloys were obtained from Ti 99.95%, Nb 99.95%, Zr 99.95% e, Ta 99.95% and Ag 99.97% as raw materials, in a vacuum arc melting furnace (VAR) ABD MRF 900. The raw materials were placed on the hearth of the water-cooled crucible in order to increase the melting points. Thus, the tantalum, which has the highest melting temperature, was placed in direct contact with the electrode, while the silver was placed as far as possible from the electrode. This approach avoided silver volatilization. The melting of the materials was carried out in an inert atmosphere of argon. The melting chamber became dark during the melting process. Decontamination of the work environment was conducted by creating a vacuum (10^−6^ bar) provided by the installation with an advanced vacuum pump. The large differences in the melting temperatures of the constituent elements (Ti-1668 °C, Nb-2477 °C, Zr-1855 °C, Ag-960 °C, Ta-3017 °C) were considered, which required the performance of several nine remelting to obtain a proper homogenization. Ingots with dimensions of d = 10 mm and L = 140–160 mm were obtained. From the as-cast ingots, we cut the cylindrical specimens with 10 mm diameter and 10 mm height. The cutting was carried out on an automatic machine with coolant and equipped with a cutting disc (Aka-Cut Ti20, AKASEL, Roskilde, Denmark), especially for titanium alloys with a hardness of 100–350 HV. Six samples were cut for each composition. The grinding and polishing of the samples were carried out on an automatic machine brand METCON Forcipol-forcimat 1 V, (Metkon, Kent, UK) using a four-step preparation kit brand AKASEL, which includes grinding discs, polishing, and polishing emulsions, which ensures a roughness below 0.2 μm. We aimed to obtain two granulations for each composition, A and B, with smooth and rough surfaces. The chemical composition of these alloys was determined from Energy Dispersive Spectroscopy (EDS). EDS was performed on five random regions for each sample, and the average value and standard deviation are presented in Table 1.

More details about the design and microstructural characterization of these alloys were presented in our previous study [27,28].

### 2.2. Surface Characterization

Atomic force microscopy (AFM) measurements were made using the XE-100 microscope from Park Systems. All AFM scans were performed in non-contact mode with NCHR sharp tips from Nanosensors™ (Neuchatel, Switzerland), with a tip radius of less than 8 nm, 125 μm mean length, 30 μm mean width, ∼42 N/m spring constant, and ∼330 kHz resonance frequency. The two-dimensional (2D) AFM images were recorded at two scales, namely (5 μm × 5 μm) and (2 μm × 2 μm), and processed with the Image Processing Program (XEI—v.1.8.0, Park Systems) for display purposes (1st order tilt correction) and roughness evaluation. Enhanced Color™—AFM images (EC-AFM) are presented based on the change in a pixel position relative to its neighbors. Below the 2D EC-AFM images, arbitrary lines (line scans collected along the X-scan direction) are displayed for each scanned sample, showing nanometric details of each investigated surface. The root-mean-square roughness (Rq) is the standard deviation of the height value in the image, and the peak-to-valley parameter (Rpv) represents the height difference between the lowest and the highest points on the surface.

The surface wettability of the samples was characterized by contact angle (θ) measurements and subsequent calculation of surface free energy (SFE). The contact angles were collected with Drop Shape Analysis System model DSA1 (FM40 Easy Drop) from KRÜSS GmbH Hamburg, Germany. The samples were put on a plane moveable table, and each drop of deionized water with a volume of 3 μL was dispensed from a micro-syringe with a stainless-steel needle (outer diameter = 0.5 mm) at room temperature. Measurements were performed in a static regime using the Young–Laplace method (Sessile Drop Fitting). For each sample, the contact angle was calculated by averaging over eight measurements on different drops.

From the contact angle data, the DSA1 program (v 1.91) can determine the solid free surface energy using the equation of state proposed by Neuman [30]. Starting with the equation of Young:

*σ_s_* = γ*_sl_* + *σ_l_* · cos*θ* (1)

where the variable γ*_sl_* is unknown, and Neuman proposed the equation [22]:

(2) $$\gamma_{\mathrm{sl}}=\sigma_{l}+\sigma_{s}-2\cdot \sqrt{\sigma_{l}\cdot \sigma_{s}}\cdot \;e^{-\beta }{\left(\sigma_{l}-\sigma_{s}\right)}^{2}$$

The symbols *σ_s_* and *σ_l_* represent the surface free energy of the solid and the surface tension of the liquid phase, respectively. γ*_sl_* represents the interfacial tension between the solid and liquid phases. *θ* is the angle between *σ_l_* and γ*_sl_* vectors, named the contact angle.

The value 0.0001247 for the constant *β* was determined empirically.

The next step was to insert the equation of Neuman in Young’s equation, and the new equation allows the calculation of the SFE with the mention that the interactions affecting the interfacial tension (disperse part and polar part of the surface tension) are neglected:

(3) $$\cos\theta =-1+2\cdot \sqrt{\frac{\sigma_{s}}{\sigma_{l}}}\cdot e^{-\beta }{\left(\sigma_{l}-\sigma_{s}\right)}^{2}$$

### 2.3. Electrochemical Characterization

The electrochemical tests were performed in a typical three-electrode cell. The measurements were carried out employing the Ag/AgCl reference electrode, platinum (Pt) counter electrode, and the samples as working electrodes using a Gamry Potentiostat/Galvanostat/ZRA model Reference 600 equipped with Echem Analyst™ software v 5.61 (Gamry Instruments Inc., Warminster, PA, USA). The samples were degreased with ethanol, washed with deionized water, and then dried in air. A PBS solution with a neutral pH of approximately 7.4 at 37 °C was used as an electrolyte to simulate the behavior of the materials in a human biological environment. The composition of the PBS solution was: Na_2_PO_4_-1.78 g/L; KH_2_PO_4_-0.24 g/L; NaCl-8 g/L; KCl-0.2 g/L. All reagents used in this study were analytical grade.

Long-term monitoring of open-circuit potential (OCP) was performed according to the static immersion method [31] in PBS solution at 37 °C for 1000 h of immersion. Electrochemical Impedance Spectroscopy (EIS) measurements were carried out at the OCP using an AC amplitude of 10 mV in the frequency range from 10^6^ Hz to 0.01 Hz, and a ZView 3.3 specialized software (Scribner Associates, Inc., Souther Pines, NC, USA) was used to fit the EIS data. To evaluate the corrosion behavior of these alloys in PBS solution, the potentiodynamic polarization was accomplished in the potential range from −0.3 V vs. OCP to 2.5 V vs. Ag/AgCl, with a scan rate of 2.5 mVs^−1^ and the corrosion parameters (corrosion potential, corrosion current, and corrosion rate) were calculated from Tafel extrapolation using Echem Analyst™ software. To prove the reproducibility of the measurements, three specimens for each type of alloy were tested in the PBS solution freshly prepared for each test.

## 3. Results and Discussion

### 3.1. Surface Roughness and Wettability

**Figure 1:**
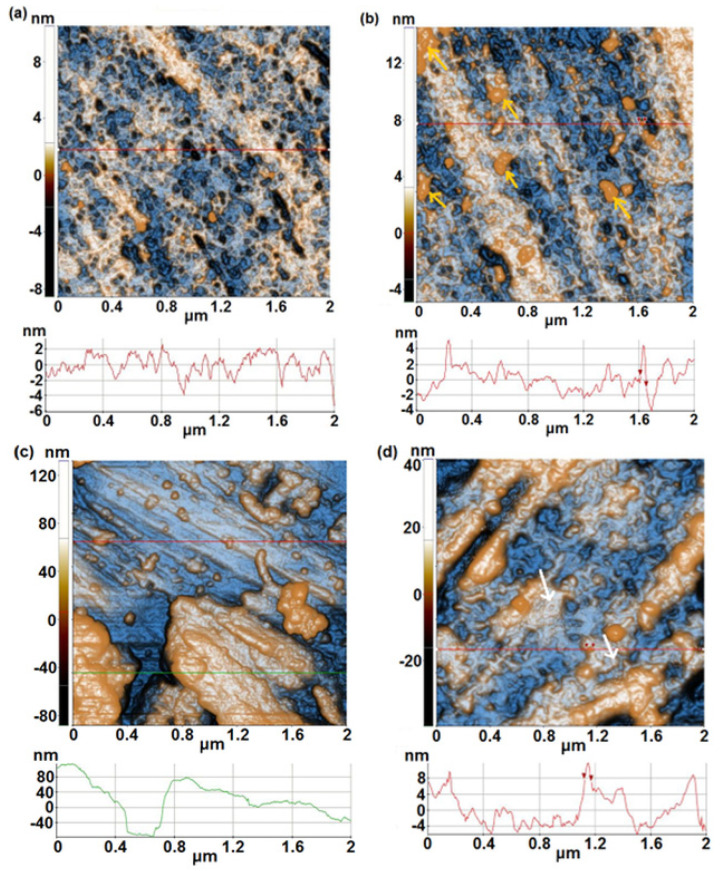
2D enhanced-contrast AFM images (topography) recorded at the scale of (2 µm × 2 µm) for sample 1A (polished) (first row), before (**a**) and after 1000 h immersion in PBS solution (**b**); 2D (enhanced-contrast) AFM images (topography) recorded at the scale of (2 µm × 2 µm) for the sample 1B (raw, unpolished) (second row), before (**c**) and after 1000 h immersion in PBS solution (**d**). Random line-scans (surface profiles) characteristic for each sample are plotted below each AFM image.

AFM technique was used to investigate the textural properties of the samples (morphology and roughness). Figure 1a shows the topography of the 1A sample (Ti20Ta9Nb8Zr2Ag) after polishing, based on a 2D AFM image scanned at the scale of (2 µm × 2 µm). The sample is flat, as suggested by the line scan plotted below the corresponding image, with a difference height level (height irregularities) of ~8 nm (from −6 to +2 nm). Random superficial pores, no deeper than 4–6 nm, are present on the scanned area, visible as dark-blue spots. The root-mean-square roughness (Rq) is 1.1 nm, while the peak-to-valley parameter (Rpv) is 19.0 nm. After immersion for 1000 h in PBS solution (Figure 1b), the 1A sample exhibits some random small deposits (indicated by orange arrows in Figure 1b), probably nucleated from the solution (small crystal salts). The roughness parameters are not much changed after the immersion experiment, as the Rq equals 1.7 nm, while Rpv was ~19.4 nm. The surface profile (line-scan) of the 1A sample after immersion (Figure 1b) exhibits a slightly smoother profile (fewer sharp peaks) in comparison with the polished surface (Figure 1a) but with the same surface difference height level of ~8 nm (from −4 to + 4 nm). Sample 1B, representing the unpolished, raw Ti20Ta9Nb8Zr2Ag alloy, appears more compact (without pores) and contains some massive materials agglomerations, tens of nm in height (Figure 1c—AFM image and corresponding line-scan). That makes the sample rough, with an Rq of 31.3 nm and an Rpv of 220.5, which are the highest values in the series of Ti20Ta9Nb8Zr2Ag alloys (as a minor observation, the upper part of the sample is less corrugated, with roughness parameter a few times lower). After immersion of 1B sample for 1000 h in PBS, it can be presumed that part of the surface irregularities is filled with the passive film (see the white arrows in Figure 1d), and the surface appears to be less corrugated—Figure 1d, as the corresponding line-scan decreases to ~16 nm (from −6 to 10 nm). Their roughness parameters are much decreased in comparison with sample 1B before immersion, as Rq equals 8.24 nm and Rpv ~79.9 nm.

**Figure 2:**
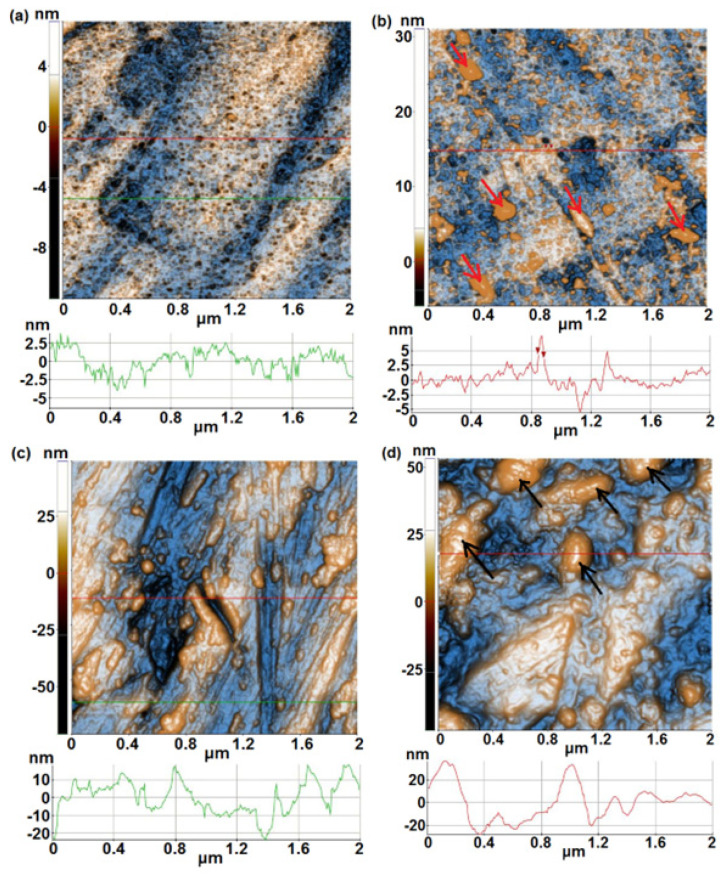
2D (enhanced-contrast) AFM images (topography) recorded at the scale of (2 µm × 2 µm) for sample 3A (first row), before (**a**) and after 1000 h immersion in PBS solution (**b**); 2D (enhanced-contrast) AFM images (topography) recorded at the scale of (2 µm × 2 µm) for sample 3B (second row), before (**c**) and after 1000 h immersion in PBS solution (**d**). Random line-scans (surface profiles) characteristic for each sample are plotted below each AFM image.

Sample 3A (Figure 2a), from Ti10Ta9Nb8Zr2Ag series of alloys, and sample 1A (from Ti20Ta9Nb8Zr2Ag series of alloys) are similar from the morphological point of view with the difference that the superficial pores (see the dark spots in the AFM image) present on sample 3A are smaller in diameter and depth. Sample 3A is also flat (Rq ~1.8 nm, Rpv ~18.3 nm), as suggested by the line scan, with a surface height difference of ~6.5 nm (from −4.0 to + 2.5 nm). After immersion in PBS, sample 3A becomes covered by different globular shaped particles (Figure 2b), separated small (few tens of nm in diameter) and agglomerated (hundreds of nm—indicated in Figure 2b by red arrows), most probably firstly attached from PBS solution and then adsorbed, part of them covering the previously observed superficial pores of sample 3A. Consequently, due to the presence of these particles, the roughness parameters slightly increased after the immersion experiment, as Rq equals 2.10 nm and Rpv ~37.0 nm. Sample 3B, before immersion (Figure 2c), shows some ditches formed, most probably during cutting (mechanical processing), a few tens of nm deep, and random material clusters with disordered shapes, leading to the following roughness parameters: Rq of 13.9 nm, and Rpv ~120.1 nm.

The line scan plotted in Figure 2c for sample 3B exhibits a vertical height level of ~30 nm (from –20 to +10 nm). Following immersion in PBS, sample 3B (Figure 2d) shows less groove, as the corresponding line scan displays a lower number of hills and valleys but massive absorbed clusters of species from the PBS solution (hundreds of nm in diameter- indicated in Figure 2d by black arrows).

It is probable that the passive film formed on the surface, as well as the adsorbed species from the solution, fill a part of the surface irregularities as the corresponding roughness parameters decrease to Rq ~13.49 nm and Rpv ~101.1 nm.

**Figure 3:**
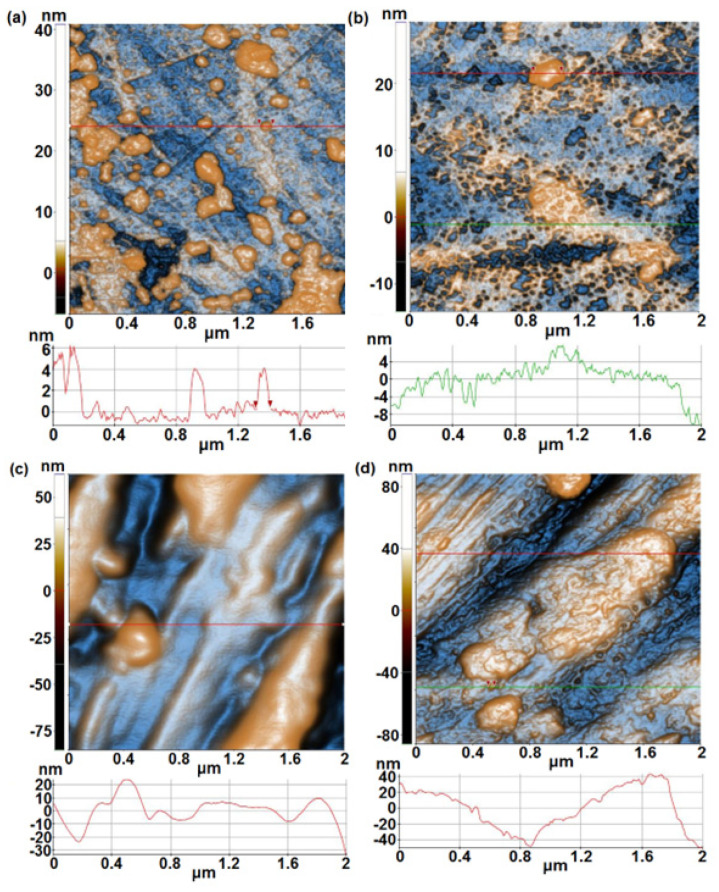
2D (enhanced-contrast) AFM images (topography) recorded at the scale of (2 µm × 2 µm) for sample 5A (first row), before (**a**) and after 1000 h immersion in PBS solution (**b**); 2D (enhanced-contrast) AFM images (topography) recorded at the scale of (2 µm × 2 µm) for sample 5B (second row), before (**c**) and after 1000 h immersion in PBS solution (**d**). Random line-scans (surface profiles) characteristic for each sample are plotted below each AFM image.

Figure 3 displays the AFM images of the alloy samples without Ta in composition (samples 5A and 5B) before and after immersion in PBS solution. Figure 3a shows the surface of sample 5A before immersion, consisting of smooth areas and randomly distributed particles, separated (around 100 nm in diameter) or gathered. The sample is relatively flat, as suggested by the corresponding line scan, with a z-height of ~7 nm (from −1 nm to +6 nm). Sample 5A is characterized by an RMS roughness of 2.4 nm and a peak-to-valley parameter of 47.6 nm (over an area of 2 × 2 µm^2^—Figure 3a). After 1000 h immersion in PBS solution, the surface acquires an aspect suggesting morphology-like corrosion (see the shape of the line scan in Figure 3b) due to the appearance of shallow pits (a few nm deep).

After immersion, sample 5A is characterized by an RMS roughness of 3.4 nm and a peak-to-valley parameter of 43.4 nm. In contrast, sample 5B displays a compact shape, with large grooves (wrinkles) leading to high values of the roughness parameters: RMS roughness of 20.0 nm and a peak-to-valley parameter of 147.5 nm. Following immersion of 1000 h in PBS solution, the morphology is changed, suggesting the adhesion of some particles from PBS solution (forming a coating-like) and some small, random shallow pits (see also the small dents along the line-scan in Figure 3d), leading to RMS roughness of 20.3 nm and a peak-to-valley parameter of 173.9 nm.

**Figure 4:**
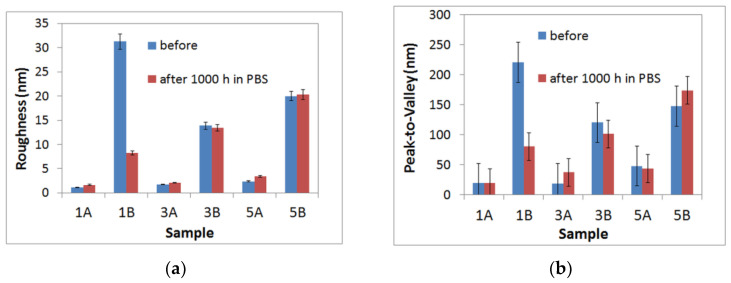
Corrugation parameters: RMS roughness (**a**) and peak-to-valley (**b**) for the samples in the initial stage (before) and after 1000 h immersion in PBS.

Figure 4 summarizes the roughness parameters [RMS roughness (a) and peak-to-valley (b)], highlighting that the pair 1A/3A is much smoother (less corrugated) than the pair 1B/3B. It can also be noted that following immersion, the roughness of the pair 1A/3A slightly increases, while in the case of 1B/3B, an opposite effect can be observed. The pair 5A/5B is similar in roughness behavior with the 1A/3A, suggesting a slight increase in corrugation after PBS immersion, as suggested by RMS roughness variation.

These results highlight the influence of the surface roughness on the formation of a passive layer containing phosphate compounds that acts as a barrier layer against corrosion processes that occur at the alloy/electrolyte interface.

As a tendency, in the case of polished samples (1A, 3A and 5A), a slight increase in roughness can be noticed following immersion for 1000h in PBS solution. On the other hand, this tendency is generally reversed in the case of the rough samples (unpolished samples), where it is presumed that the passivation process (the film formed) diminishes the surface defects (by filling the previous pits and valleys).

The surface wettability of metallic biomaterials is an important parameter in affecting biological reactions (i.e., protein adsorption and cell adhesion), which can be investigated by contact angle measurement [32]. It is well known that hydrophilic surfaces have better cell adhesion than hydrophobic ones. Higher wettability, and thus lower contact angle, favor cell adhesion to biomaterial surfaces, which is important in the case of orthopedic implants, as it influences the process of tissue-implant integration. On the other hand, lower wettability—and thus a higher value of contact angle, is vital in the case of such clinical applications (for example, elements of cardiac valves or dialysis devices), where the lowest protein absorption is desired [33].

The contact angle measurements performed with distilled water on all sample surfaces are illustrated in Table 2. For all samples with tantalum in chemical composition, the contact angle highlights the hydrophilic behavior of the surface after mechanical polishing (i.e., the contact angle is smaller than 90°). It is well known that the presence of Ta in the chemical composition of the alloys might impact the surface’s wettability because Ta presents more hydrophilicity than Ti [34]. The hydrophilic character of the alloy surfaces is preserved for all samples, even after 1000 h of immersion in PBS solution. At the samples without Ta in chemical composition (samples 5A and 5B), an almost hydrophobic surface can be observed with a contact angle value of 86.2 ± 4.12 (5A sample) and 82.5 ± 1.49 (5B sample). After 1000 h of immersion in PBS, the contact angle values decrease, and the sample 5B with a rough surface shows the lowest value of 67.3 ± 5.49.

The contact angle values illustrated in Table 2 suggest the hydrophilic character of the surface of all samples after 1000 h of immersion in PBS. Furthermore, a decrease of up to 18 degrees in contact angle values is noticed for 1B and 3B samples, suggesting higher hydrophilicity of the surfaces. These low values of contact angles (i.e., 47.74 ± 5.31 for 3B and 54.3 ± 2.82 for 1B samples) could be due to the difference in the topography of the surfaces (1B, 3B have larger roughness than 1A, 3A), as well as, to the possible presence of a more hydrophilic group (-OH) on the surface. According to literature reports, [19,20] the chemistry of the surface is the main issue that generates a better wettability of the surface, whilst a relatively smooth surface, which prevents the condensation of water droplets and surface wettability, causes an increase in contact angles. Moreover, it is known that oxidized titanium surfaces show a high wettability with increasing roughness [35]. The results presented in Table 2 for all samples exhibited hydrophilic surfaces that promote the interactions with biofluids, cells, and tissue and give rise to better biocompatibility [36]. It is well known that cell adhesion is correlated with roughness parameters, particularly with parameters describing the organization of the surface roughness. Several studies [37] using immuno-histochemical methods have shown the presence of actin and vinculin in cultured osteoblasts on various biomaterials (i.e., Ti6Al4V, CoCrMo) and have noticed that osteoblast attachment was greater on Ti6Al4V with various degrees of roughness because cell spreading and cytoskeletal organization were enhanced. In addition, reported data [37] revealed that the distribution of focal contacts shows the mode of adhesion of osteoblasts on the different roughness. On smooth surfaces, focal contacts are distributed uniformly on all the membrane surfaces that are in contact with the substratum, whilst on rough surfaces, focal contacts are visible only at the extremities of cells [16].

Another factor that influences the surface wettability is the surface free energy (SFE) of the solid-liquid interface. When the contact angle is measured on a surface, the water’s tendency to bond is determined by the SFE of the surface that makes contact with water [14]. The alloys’ surface free energy (SFE) increases with the decrease in contact angle surface, making it more hydrophilic [14,15,20]. The SFE and the contact angle are irreversibly related. Table 2 summarizes the surface free energy of all samples estimated before and after immersion in PBS. There are no significant differences in SFE values for smooth and rough surfaces. After immersion, the SFE values of the samples increase because the contact angles decrease, as well as considering the changes in the chemistry of the surface brought by the formation of an oxide layer on the surface [14,20].

Concerning the relationship between corrosion resistance and SFE, some studies have pointed out that alloys with low SFE exhibit high corrosion resistance [2]. In this view, one may assume that our samples show comparable corrosion resistance because, as wettability investigations show, they exhibit close SFE values (see Table 2).

### 3.2. Electrochemical Characterization

The electrochemical evaluation of the samples, without and with two Ta concentrations in chemical composition and different grades of roughness, was performed for long-term (1000 h) immersion in PBS solution at 37 °C to highlight the effect of the roughness surface and the Ta amount on corrosion resistance of these alloys. The main electrochemical parameters (*E_oc_*, *E_corr_*, *R_corr_*, *R_p_*) that are considered in this evaluation were estimated from the potential measurements in open circuit (OCP), the electrochemical impedance spectra (EIS), the potentiodynamic polarization curves and Tafel extrapolation.

#### 3.2.1. Long-Term Monitoring of the Open Circuit

**Figure 5:**
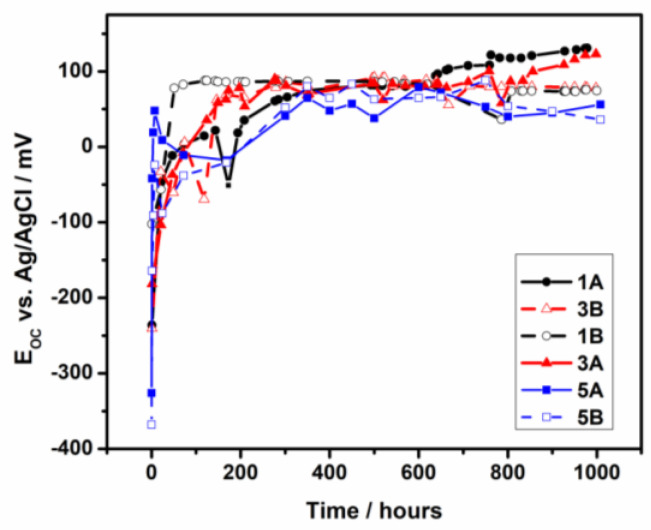
OCP evolution of 1A, 3A, 5A, 1B, 3B and 5B samples in PBS solution during 1000 h of immersion at 37 °C.

Figure 5 illustrates the evolution of the open circuit potentials, *E_oc_* of all samples in PBS at 37 °C for 1000 h of immersion.

During the first 100 h of immersion, the *E_oc_* values of all samples increase sharply and achieve steady with the extending immersion time. Between 100 and 200 h of immersion, a slight decrease in *E_oc_* for all samples is evidenced. This behavior could be due to the activation of the surface due to the dissolution of the native oxide film that grows on the surface. No more differences between the *E_oc_* values of samples 1A, 3A, and 5A were observed; all samples have had close behavior throughout the immersion period, with a slow increase in *E_oc_* for the 1A sample after 600 h of immersion. All three samples keep their *E_oc_* values between +50 and +100 mV vs. Ag/AgCl, suggesting that a passive film was formed on the surface. As regards the influence of the roughness surface on the *E_oc_* values, all three samples with rough surfaces (1B, 3B and 5B) revealed similar behavior; *E_oc_* accomplished to be steady with the extending immersion time, suggesting the enhancement in the stability of the passive films formed on the surface. *E_oc_* values are distributed between −150 mV and +150 mV vs. Ag/AgCl for 1A, 3A and 5A polished samples and between −250 mV and + 75 mV vs. Ag/AgCl for 1B, 3B and 5B rough samples. All *E_oc_* values are placed in the passive potential range for titanium and tantalum on the Pourbaix diagrams [38]. Some fluctuations of *E_oc_* values noticed during the period of immersion could be due to the adsorption or/and dissolution processes that are occurring at the interface: the adsorption of phosphate ions on the surface of the samples, followed by the partial dissolution of the complexes formed with the components of film grown on the surface, is a possible answer. As literature reports [39], the passive film could be a balanced state of formation and dissolution in a natural long-time immersion test.

After 1000 h of immersion, the stabilized *E_oc_* values of all samples correspond to the presence of a passive film on the surface that provides protective properties against corrosion in PBS.

#### 3.2.2. Long-Term Corrosion Evaluation

**Figure 6:**
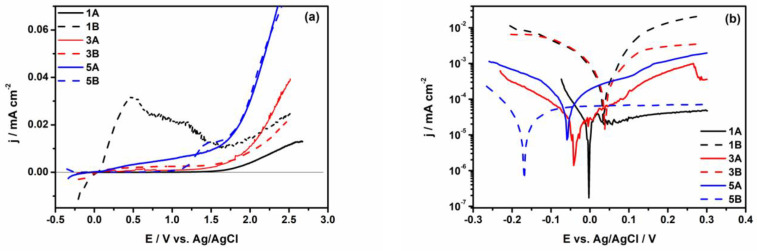
The potentiodynamic (**a**) and Tafel curves (**b**) of 1A, 3A, 5A, 1B, 3B, and 5B samples after 1000 h of immersion in PBS solution at 37 °C.

Figure 6a, b show the polarization and Tafel curves performed after 1000 h of immersion in PBS solution for all studied samples (1A, 3A, 5A and 1B, 3B, 5B).

As potentiodynamic curves depict (Figure 6a), the alloys with Ta amount in composition and smooth surface (1A and 3A samples) present low values of current density on a large region of potential, from 0.0 V to 1.7 V vs. Ag/AgCl, suggesting the passive character of the film grown on the surface during long-time immersion (j_pass_. ≤ 1 µA cm^−2^), and a slow increase in passive current (10 µA cm^−2^) was observed up to 2.0 V for 3A sample and to 2.5 V for 1A sample. Furthermore, up to 2.5 V, no breakdown or pitting occurred for these samples. For alloy without Ta content in composition (5A sample), a slight increase in current density is clearly visible in Figure 6a, as well as a diminution of the passivation range, up to 1.55 V vs. Ag/AgCl. Over this potential, a significant increase in passive current density (j_pass_ = 70 µA cm^−2^) is noticed, suggesting low protective properties of the passive film grown on the 5A sample’s surface. Besides, it is interesting to note the different behavior of the 1B sample, where a rapid increase in the current density up to 30 µA cm^−2^ on the anodic polarization curve is observed. A moderate decrease in the current density (from 30 µA cm^−2^ to 10 µA cm^−2^) is remarked in the potential range between 0.5 V and 1.6 V, followed by a slight increase of about 20 µA cm^−2^ at 2.5 V. This response could be due to a transpassive process that occurs on the surface of 1B sample probably due to the presence of numerous defects onto the surface that led to the formation of a defective passive layer [25], and a higher roughness in comparison with samples 3B and 5B according with the AFM measurements. These results are in line with reported data that revealed the direct relation between current density and surface roughness [40]. In addition, a small diminish of the passive potential range was also evidenced for the 5B sample, which presents a short transpassive range (from 1.2 V to 1.7 V), and a significant increase in current density similar to the 5A sample is identified over 1.7 V.

As AFM images show (Figure 3 and Figure 4), the surface of the 1B sample appears more compact with massive material agglomerations that make the sample rougher than the 3B and 5B samples, and a larger surface area is developed. Thus, a larger surface area in contact with the PBS solution is gained for the 1B sample, leading to an increase in the current density. Consequently, a decrease in the rate of passivation is attained.

Figure 6b illustrates the Tafel curves recorded after 1000 h of immersion in PBS. It is evidenced from Tafel curves that the *E_corr_* value for all samples moves towards a more positive direction, especially for samples with rough surfaces and Ta amount in composition. This is in agreement with the evolution of the OCP. This behavior reveals a passive character of the surface samples in PBS solution. Additionally, the main electrochemical parameters, corrosion potential (*E_corr_*), corrosion current density (*j_corr_*), corrosion rate (*R_corr_*), and polarization resistance (*R_p_*) were estimated by Tafel extrapolation and are presented in Table 3.

For 1B and 3B samples, more positive values of corrosion potential (*E_corr_*) were noticed over time, revealing a passive film formation, whereas for 1A, 3A, 5A, and 5B samples, a negative value of *E_corr_* was observed. Because no significant differences in *E_corr_* values are noted at 1A, 3A, and 5A samples, no reliable information about corrosion behavior might be obtained from this corrosion parameter.

The linear anodic polarization curves recorded for all samples and illustrated in Figure 6a highlight a significant difference between sample 1B and the other samples in terms of passivation. An important increase in the corrosion current density is also observed for the 1B sample (i.e., 107 ± 0.15 nA cm^−2^) compared with the 1A sample (17.2 ± 0.02 nA cm^−2^), even though they have the same chemical composition but different surface roughness. This suggests that the film formed on the surface of the 1B sample is not stable or continuous. After 1000 h of immersion for samples 1A and 3A, the lowest values of *i*_cor_ were estimated (i.e., 17.2 ± 0.02 nA cm^−2^ and 53 ± 0.11 nA cm^−2^), suggesting a high corrosion resistance in PBS solution. Furthermore, both 1A and 3A samples exhibited a large potential range (>2.0 V) where the current density is a passive one (j < 10 µA cm^−2^). Additionally, they showed low corrosion rate values (*R_corr_* < 1 µm y^−1^), indicating that the protective film formed on the surface of these samples remains stable over time.

A slight increase in the corrosion rate (between 1.35 ± 0.1 and 3.01 ± 0.25 µm y^−1^) and a small narrowing of the passive potential range were observed for all samples with rough surfaces (1B, 3B, 5B) after 1000 h of immersion in PBS. Consistent with the corrosion parameters estimated after 1000 h of immersion in PBS, the best corrosion performances are shown by the 1A and 3A samples.

**Figure 7:**
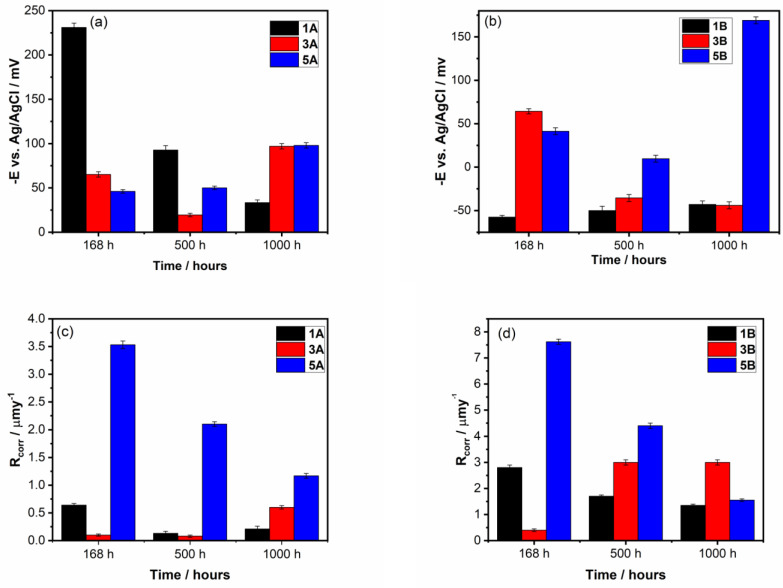

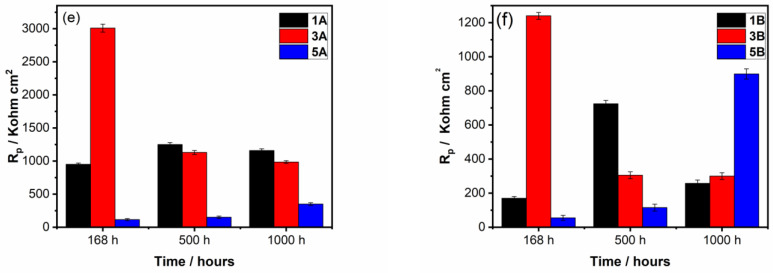
Long time evolution of corrosion potential (**a**,**b**), corrosion rate (**c**,**d**), and polarization resistance (**e**,**f**) of all samples in PBS solution at 37 °C.

According to the standard resistance classes [41], the Ti20Ta9Nb8Zr2Ag and Ti10Ta9Nb8Zr2Ag alloys with smooth surfaces (1A and 3A samples) are considered to be perfectly stable materials, exhibiting the best corrosion performance during long-term immersion in PBS (with a corrosion rate < 1 µm y^−1^). On the other hand, the alloys without Ta in their composition and all samples with a rough surface are classified as stable resistance class materials, with a corrosion rate ranging from 1.35 ± 0.1 µm y^−1^ to 3.01 ± 0.25 µm y^−1^. The long-term evolution of the electrochemical parameters (*E_corr_*, *R_corr_*, *R_p_*), which are important for assessing the corrosion performance of the Ti-Ta alloys studied in PBS at 37 °C, is illustrated in Figure 7. A comparison between the samples with smooth surfaces (1A, 3A, and 5A) and rough surfaces (1B, 3B, and 5B) is also highlighted.

Firstly, after immersion of the samples for 168 h, it was observed that the *E_corr_* of the samples with smooth surfaces (1A, 3A) was more negative than that of the samples with rough surfaces (1B, 3B). However, over time, *E_corr_* tends to increase towards less negative values (Figure 7a). These results are surprising because it is well known that a smooth surface usually improves the corrosion performance of the materials [42]. A different behavior is observed for samples 5A and 5B, without Ta amount in composition, both samples revealing negative values of *E_corr_* throughout the immersion period (Figure 7b). Long-time evolution is unfavorable for samples 5A and 5B, the corrosion potential became slightly more active, and the current density increases were probably generated by the dissolution–repassivation processes at the interface between the samples and PBS [43]. Moreover, adsorption of the PO_4_^3−^ ions on the sample surface, acting on a barrier layer between the sample surface and PBS, leads to an increase in the film thickness. 

According to the literature report [24,44], the process of adsorption of phosphate ions is fast, but the surface became saturated after a long time of immersion in the solution. This fact could be an important factor that causes changes in the corrosion parameters of the studied alloys during the immersion period.

Secondly, the corrosion rate (*R_corr_*) and polarization resistance (*R_p_*) should be analyzed together because both parameters are undoubtedly associated with the corrosion resistance of the alloys. As Figure 7c,d illustrate, the corrosion rates of 1A and 5A samples slowly decrease, whilst the 3A sample shows a minor increase.

It is noticed that the 3A sample shows the highest *R_p_* value after 168 h of immersion, indicating that a native passive film is formed on the surface. However, over time (1000 h), a significant decrease in *R_p_* with a small increase in *R_corr_* (from 0.1 to 0.6 µm y^−1^) is observed.

Polarization resistance *R_p_*, which characterizes the passive film resistance, exhibits higher values for 1A, 3A, and 5A samples in comparison with 1B, 3B, and 5B samples, as shown in the histograms depicted in Figure 7e,f. The corrosion rate (*R_corr_*) values of 1B, 3B, and 5B samples, estimated by Tafel extrapolation, are two to four times higher than that of 1A, 3A, and 5A samples, and are in line with the noticeable decrease in *R_p_* values. This significant increase in *R_corr_* for the samples with the rough surfaces can be explained by a large surface development on 1B, 3B, and 5B samples in comparison with smooth ones (1A, 3A, and 5A), given that an irregular surface rough, it is more susceptible to oxidation, and the film formed will be easier deteriorated by aggressive biofluids, originating the corrosion process [42,45]. Moreover, some depassivation zones can appear on the samples with rough surfaces because of the presence of numerous defects on the surface, which lead to the formation of a defective passive film [25,46]. This assumption follows the AFM analysis that exhibits morphological changes in the samples with rough surfaces, revealing large grooves leading to high values of the roughness parameters (5B sample) and many irregularities due to the adsorbed species on the surface (1B and 3B samples).

The corrosion parameters are quite closer or even superior to those reported in the literature for similar Ti-Nb-Zr-Ta alloys in different biofluids (Table 4). 

These findings point out that the corrosion performance of the studied alloys is comparable with that reported in the literature. Moreover, as can be seen in Table 4, the corrosion performance of the alloys containing Ta and with smooth surfaces (i.e., 1A and 3A) is by far better than that reported for other alloys.

In conclusion, all samples exhibit good corrosion protection in PBS for long-time immersion. The best corrosion resistance was observed for the samples with smooth surfaces and with 10% or 20% Ta. For the samples with or without Ta in their composition and with a certain roughness, a lower corrosion performance was noticed. These results appear to attest that only the presence of Ta in alloy composition, which should bring about good stability of the film [50], does not necessarily improve the protective properties of the passive film. Actually, both the presence of Ta in alloy composition and a smooth surface provides good corrosion performance of these alloys. This behavior most probably resides in the presence of Ta_2_O_5_ and TiO_2_ in the film composition and in the lower accessibility of the fluid to the alloy surface [8,23,50].

It is well known that at a rough surface, a higher contact surface/fluid occurs, likely yielding to a surface that is more susceptible to corrosion attack. These results are supported by EIS investigations, which revealed that the alloys with smooth surfaces and a certain amount of Ta (1A and 3A samples) have high resistance of the film (i.e., R_2_ is 5.673 MΩ cm^2^ and 3.480 MΩ cm^2^ for 1A and 3A samples).

#### 3.2.3. Electrochemical Impedance Spectroscopy

**Figure 8:**
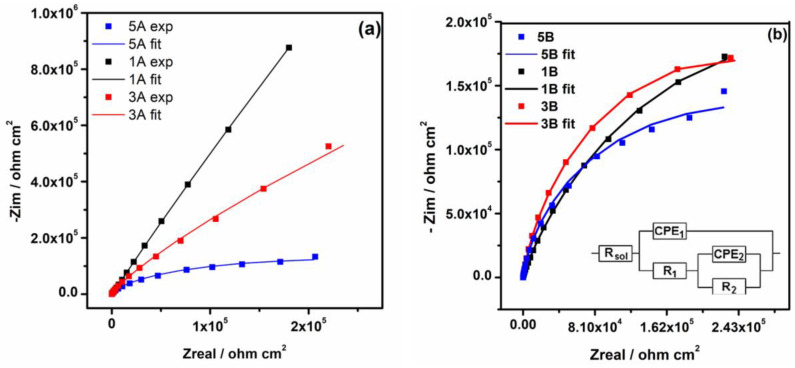
Nyquist plots of 1A, 3A, 5A samples (**a**), 1B, 3B, and 5B (**b**) samples after 1000 h of immersion in PBS at 37 °C. In set: Electric equivalent circuit.

To evidence the changes at the passive film/electrolyte interface upon exposure of all samples to PBS solution, the EIS investigations were performed simultaneously with open circuit potential monitorization. The EIS data recorded after 1000 h of immersion are represented in Nyquist plots (Figure 8). A single capacitive arc characterized by a large, depressed semicircle is observed in Nyquist plots for 1A and 3A samples, suggesting a similar passivation mechanism. Regarding the Nyquist plot for the 5A sample, a decrease in the diameter of the semicircle is noticed (Figure 8a), indicating a different passivation process. Literature reports have shown that a larger capacitive loop diameter is related to the dielectric properties of the oxide film formed on the metal surfaces and corresponds to higher corrosion resistance [51,52]. From the Nyquist plots recorded for all samples, it was evidenced that the shape of these plots changes from a linear one for 1A and 3A samples to an arc shape for the others (Figure 8b), suggesting that at samples 1A and 3A a mass transfer of chemical species from PBS to the samples occurs [53].

To further analyze the corrosion behavior of all samples in PBS solution, the EIS spectra were fitted with an electric equivalent circuit (EEC) (see depicted EEC in Figure 8) with two-time constants that correspond to an inner thin layer (CPE2 and R2) associated to the formation of the passive film on the alloy surface that is of great importance for hindering the corrosion processes and an outer layer (CPE1 and R1) associated to the adsorption/desorption processes at the interface alloy/electrolyte during the immersion. The Chi-squared value (X^2^) is less than 2 × 10^−3^ for all fitted samples, suggesting that this circuit fits our experimental data well (see Figure 8). Considering the heterogeneity of the surface and the complex physical phenomena that take place, a constant phase element instead of capacitance is more suited for fitting the EIS spectra. The Chi-squared value (X^2^) is less than 2 × 10^−3^ for all fitted samples, suggesting that this circuit fits our experimental data well. The CPE is a constant phase element, which consists of a capacitance (C) and a deviation parameter *n*, which describes the deviation of the ideal capacitive behavior of the passive film attributed to the roughness and the defects on the surface samples. CPE_1_ and CPE_2_ are described as capacitances of the outer and the inner layer.

We consider that in our case, the parameter that gives insights into the corrosion performance of these samples is the resistance of the inner layer (R_inner_) because it represents the resistance of the passive layer formed on the alloy surface [29,54].

From the fitted results, one may estimate that the R_2_ of all samples with smooth surfaces are of the order of MΩ cm^2^, pointing out that the passive film formed on these types of surfaces has good corrosion resistance. However, the resistance of the inner layer of the 1A sample, i.e., 5.673 MΩ cm^2^, is 1.6 times higher than that of the 3A sample, i.e., 3.480 MΩ cm^2^, and 5.7 times higher than that of the 5A sample, i.e., 1.001 MΩ cm^2^, suggesting that the passive protective film formed on 1A and 3A samples has remarkable protective properties.

In contrast, a non-negligible decrease in the inner layer resistance (R_2_) is observed for the samples with rough surfaces, i.e., 437 kΩ cm^2^, 449 kΩ cm^2^ and 446 kΩ cm^2^ for 1B, 3B and 5B samples, respectively. A possible explanation for this decrease could be due to a large contact surface/fluid at the samples with rough surfaces, which could yield a lower corrosion resistance [32].

In conclusion, one may consider that samples with a certain amount of Ta in their composition and with a smooth surface exhibit a higher impedance associated with a lower susceptibility to corrosion in PBS solution after a long time of immersion. These EIS results are in good agreement with OCP evolution and potentiodynamic.

## 4. Conclusions

This study investigated the influence of surface roughness and a certain amount of tantalum on the corrosion performance of the TiTa9Nb8Zr2Ag alloys after long-term immersion in PBS at 37 °C, and the results obtained are summarized as follows:

1. From AFM investigations before immersion, it was noticed that the 1A, 3A, and 5A samples show smooth surface, whereas the 1B, 3B and 5B samples show rough surface. After immersion, a similar trend is observed, in spite of the fact that a significant decrease in surface roughness is evidenced in the 1B sample.
2. The moderate values of the contact angles were observed for all samples (i.e., from 49.1° to 70.8° for 1A, 3A, and 5A samples, and from 54.3° to 67.3° for 1B, 3B, and 5B samples), suggesting the hydrophilic character of the surface of all samples which is expected to have a beneficial effect on the osseointegration process.
3. After 1000 h of immersion in PBS, the electrochemical results revealed that 1A and 3A samples have excellent corrosion performance, i.e., 0.21 < *R_corr_* < 0.6 µm y^−1^, pointing out that from a corrosion point of view, they are perfectly stable materials. However, the other samples (5A, 1B, 3B, and 5B) might be considered stable materials because a low corrosion rate is noticed (1.35 < *R_corr_* < 3.01 µm y^−1^). Based on OCP evolution, this excellent corrosion behavior observed at 1A and 3A samples results from forming a stable passive film with remarkable protective properties. Actually, the synergetic effect of tantalum in alloy composition and of a smooth surface is responsible for the outstanding corrosion resistance of 1A and 3A samples. These findings are supported by the evolution over time of the corrosion parameters estimated for these samples.
4. Based on these findings, one may presume that Ti20Ta9Nb8Zr2Ag and Ti10Ta9Nb8Zr2Ag alloys with smooth surfaces are most suited from a corrosion point of view for medical applications because both *R_corr_* is low (0.21 < *R_corr_* < 0.6 µm y^−1^) and ΔE_pass_ is large (2.05 V < ΔE_pass_ < 2.4 V). These results are also supported by EIS data, which attested that the resistances associated with the passive film (R2) are the highest (i.e., 5.6 MΩ cm^2^–3.4 MΩ cm^2^) for these two samples.

## Figures and Tables

**Table 1:** The chemical composition (% wt.) of the main components of the tested alloys.

Alloy	Sample	% Ti	% Ta	% Nb	% Zr	% Ag
Ti20Ta9Nb8Zr2Ag	1A, 1B	Balance	20.2 ± 0.6	9.0 ± 0.1	7.8 ± 0.1	1.9 ± 0.1
Ti10Ta9Nb8Zr2Ag	3A, 3B	Balance	8.9 ± 0.3	10.2 ± 0.6	7.9 ± 0.3	1.9 ± 0.1
Ti9Nb8Zr2Ag	5A, 5B	Balance	0	9.2 ± 0.4	8.2 ± 0.2	1.8 ± 0.2

**Table 2:** The contact angle and surface free energy (SFE) values were performed before and after 1000 h of immersion in PBS solution at 37 °C.

Sample	θ [°] (Before Immersion)	θ [°] (After Immersion)	SFE [mN/m] (Before Immersion)	SFE [mN/m] (After Immersion)
1A	74.3 ± 2.09	62.6 ± 3.65	38.68 ± 0.89	45.87 ± 2.21
1B	71.6 ± 1.52	54.3 ± 2.82	40.35 ± 0.94	50.84 ± 1.66
3A	67.2 ± 0.48	49.1 ± 3.95	43.07 ± 0.29	53.93 ± 2.27
3B	64.7 ± 2.65	47.7 ± 5.31	44.80 ± 2.11	55.82 ± 3.17
5A	86.2 ± 4.22	70.8 ± 4.12	31.27 ± 2.63	40.85 ± 2.54
5B	82.5 ± 2.49	67.3 ± 5.49	33.58 ± 1.55	43.00 ± 3.37

**Table 3:** Corrosion parameters of TixTa9Nb8Zr2Ag alloys after 1000 h of immersion in PBS at 37 °C.

Sample	*E_corr_*, mV	*J_corr_*, nA cm^−2^	*R_corr_*, µm y^−1^	*R_p_*, kΩ cm^2^	Δ*E_pass_*, V
1A	−33 ± 1	17.2 ± 0.02	0.21 ± 0.01	1160 ± 2.5	2.4 ± 0.05
1B	43 ± 2	107 ± 0.15	1.35 ± 0.1	257 ± 1.0	-
3A	−97 ± 5	53 ± 0.11	0.6 ± 0.05	456 ± 1.5	2.05 ± 0.02
3B	44 ± 2	228 ± 0.24	3.01 ± 0.25	305 ± 1.5	2.04 ± 0.02
5A	−98 ± 5	134 ± 0.18	2.1 ± 0.2	151 ± 1.0	1.65 ± 0.01
5B	−169 ± 5	68.6 ± 0.15	1.55 ± 0.1	899 ± 2.0	1.62 ± 0.01

**Table 4:** Corrosion parameters of Ti-based alloys in different biofluids.

Sample		*E_corr_* V	*J_corr_* µA cm^−2^	*R_p_* kΩ cm^2^	*R_corr_* µm y^−1^	Biofluid	Reference
Ti_42.5_Zr_42.5_Nb_5_Ta_10_		−0.057	0.041	-	-	SBF	[15]
Ti_30_Ta_3_Ag		−0.369	0.143	-	-	Ringer’s	[34]
Hf_27_Nb_12_Ta_10_Ti_23_Zr_28_		−0.433	0.19	35	-	AS	[47]
		−0.463	0.21	98	-	SBF	
Ti_35_Nb_25_Zr_25_Ta_15_		−0.28	0.10	-	9.62	Ringer’s–Hartmann’s	[13]
Ti_42.5_Zr_42.5_Nb_5_Ta_5_Mo_5_		−0.336	0.030	-	-	PBS	[48]
Ti_27.78_Zr_27.78_Hf_27.78_Nb_8.33_Ta_8.33_		−0.437	0.122	-	0.93	Hank’s	[49]
Ti20Ta9Nb8Zr2Ag	1A	−0.033	0.0172	1160	0.21	PBS	Our work
	1B	0.043	0.107	257	1.35		
Ti10Ta9Nb8Zr2Ag	3A	−0.097	0.053	456	0.6		
	3B	0.044	0.228	303	3.01		
Ti9Nb8Zr2Ag	5A	−0.098	0.134	151	2.1		
	5B	−0.169	0.0686	899	1.55		

## Data Availability

The original contributions presented in the study are included in the article, further inquiries can be directed to the corresponding authors.

## Nomenclature

Parameter Notation	Parameter Definition
1A	Ti20Ta9Nb8Zr2Ag smooth surface
3A	Ti10Ta9Nb8Zr2Ag smooth surface
5A	Ti9Nb8Zr2Ag smooth surface
AFM	Atomic force microscopy
1B	Ti20Ta9Nb8Zr2Ag rough surface
3B	Ti10Ta9Nb8Zr2Ag rough surface
5B	Ti9Nb8Zr2Ag rough surface
C	Capacitance, F/cm^2^
CPE	Constant phase element, s^n^ Ω^−1^ cm^−2^
CPE_1_	Constant phase element outer layer, s^n^ Ω^−1^ cm^−2^
CPE_2_	Constant phase element inner layer, s^n^ Ω^−1^ cm^−2^
E_corr_	Corrosion potential, mV
EEC	Electric equivalent circuit
EIS	Electrochemical impedance spectroscopy
E_OC_	Potential open circuit, mV
i_corr_	Corrosion current, A
j_corr_	Corrosion current density, A cm^−2^
OCP	Open circuit potential
PBS	Phosphate buffer solution
PP	Potentiodynamic polarization
R_1_ = Rout	Resistance out layer, Ωcm^2^
R_2_ = Rinner	Resistance inner layer, Ωcm^2^
R_corr_	Corrosion rate, µm y^−1^
RMS	Root-Mean-Square roughness, nm
Rp	Polarization resistance, Ω cm^2^
Rsol	Solution resistance, Ω cm^2^
Rpv	Peak-to-valley, nm
Rq	Roat-mean square, nm
SFE	Surface free energy, mN/m
Ɵ	Contact angle, degrees
Ϭ_1_	Surface tension of the liquid, mN/m
Ϭ_s_	Surface free energy of the solid, mN/m
